# Reviewer Acknowledgments

**DOI:** 10.13023/jah.0401.08

**Published:** 2022-02-13

**Authors:** F. Douglas Scutchfield, Charlotte S. Seidman, Robert M. Shapiro

**Affiliations:** College of Public Health, University of Kentucky; Seidman Writing; SEAHEC Medical Library

**Keywords:** Appalachia, reviewers, peer review, acknowledgment

## Abstract

We offer these reviewers our heartfelt thanks for a task that usually goes unrewarded in the academic environment. This year, we are especially grateful for those people listed below. They have assisted us in reaching the start of our 4th year, by guiding our decisions with your knowledge and capabilities.

As with all peer-reviewed medical journals, the *Journal of Appalachian Health* could not be the credible online journal it is without the help of the competent content experts we choose to review each of our research, review, and special articles.

And each year, we offer them our heartfelt thanks for a task that usually goes unrewarded in the academic environment. This year, we are especially grateful for those people listed below. They have assisted us in reaching the start of our 4th year, by guiding our decisions with your knowledge and capabilities.

A. Brianna Sheppard

Aaron Kruse-Diehr

Adam Hege

Al Delia

Alan M Ducatman

Alisha Farris

Allison Lastinger

Allison Ross

Altaf Saadi

Amy A. Eyler

Ana Contardo

Andrew C. Rucks

Anita Chandra

Anna G. Hoover

Anne Julian

Arthur L Frank

Asher Schranz

Austin Brown

Betsy Aumiller

Bonnie J. Hackbarth

Bradford W. Hesse

Brandy McCann

Brenna Kirk

Brent Emerson

Bria Gresham

Bud Nicola

Cameron Lanier

Carol L. Steltenkamp

Carolann Curry

Cecily Fassler

Chris Prener

Christina Rosen

Christine Schalkoff

Janice Vick

Jannine Bailey

Christopher Seitz

Cooper Johnson

Craig G Gundersen

Danelle Stevens-Watkins

Daniel Brook

Daniel Yoder

Danielle Nunnery

Dannell Boatman

David Logan

Deborah Azrael

Dennis McCarty

Elisa Rodriguez

Elizabeth Shay

Eman Tadros

Emily B Zimmerman

Emily Guseman

Erin Bouldin

Erin N Haynes

Everett Magann

Gary Cuddeback

Grace Gorenflo

Gretchen Ely

Gulzar H. Shah

Heather M Bush

Helena Vonville

Hilary L. Surratt

Hollie Pellosmaa

Ingrid E Luffman

J K Ibrahim

Jade Zimpfer

Janet Bronstein

Janet Mullins

Janice Dixon Key

Linda Alexander

Linda G. Kean

Jenine K Harris

Jennie L Yoost

Jennifer A Mallow

Jennifer B Shinn

Jennifer Pooler

Jennifer Tyson

Jerod Stapleton

Jill Crainshaw

Jill D. Moore

Jill Krueger

Jill Nolan

Joanne Patterson

Joe Bohn

Joel Segel

Jonathan C. Young

Judith Beverly

Julie Marshall

Julie Sakowski

Julie Townsend

Kandace McGuire

Karen Caldwell

Kate E Beatty

Kate Leger

Katherine A Jones

Katherine Ross

Kathi Harp

Kathleen C Brown

Kathleen Winter

Kelly Blake

Kelly Brunst

Kenneth Daniel Ward

Ketrell McWhorter

Kevin A. Pearce

Kimberly Kelly

Kostas Skordas

Kyle Thompson

Laura Downey

Laura McArthur

Laura R. Lander

Lauren Andersen

Laurie L Meschke

Laurie Theeke

Rich Sutphin

Richard N. Greenberg

Richard W. Christiana

Rick Slaven

Robert Heimer

Makenzie Barr

Marc Kiviniemi

Mark Dignan

Martha L Buchanan

Mary Beth Babos

Mary Davis

Matthew F Hudson

Matthew L Bush

Matthew Miller

Maureen Byrnes

Maureen P Bezold

Melissa D Gutschall

Melissa Slone

Meredith S. Duncan

Michael E Thomas

Michael Fisher

Michael Meit

Michael Rozier

Michael Topmiller

Michele Morrone

Mildred Maisonet

Mira Katz

Nadya Belenky

Nafisa Jadavji

Nancy Carlson

Nicholas Newman

Olga Khavjou

Patricia B Wright

Paul Erwin

Paul Joudrey

Peg Allen

Philip M. Westgate

Rachel R Walden

Rachel R Walden

Rachel Vickers-Smith

Raeven Chandler

Raj Balkrishnan

Randolph F. Wykoff

Raynor Mullins

Rebecca Adkins Fletcher

Rebecca Reece

Reda Wilson

Stephanie Boone

Stephanie Ettinger De Cuba

Stephanie Jilcott-Pitts

Stephanie Moore

Steve Browning

Robert M. Shapiro II

Robin Vanderpool

Robyn Goodman

Roger L Blackwell Jr

Samantha M Harden

Samuel Matheny

Sarah Brothers

Sarah E. Nelson

Sarah Raskin

Sarah Sofka

Scott Langevin

Scott P. Hays

Scottie B. Day

Seth Himelhoch

Snow Xue Feng

Stephanie Bonnes

Steve M. Davis

Tauna Gulley

Ted Raybould

Terry L. Bunn

Thomas Bodenheimer

Thomas Ricketts

Timothy A Joyner

Tina Antill Keener

Umedjon Ibragimov

Va Nee Van Vleck

Virginia Valentin

W. Jay Christian

Wayne Coombs

Wendy Braund

William Copeland

Yilin Yoshida

